# Systems biology analysis of publicly available transcriptomic data reveals a critical link between *AKR1B10* gene expression, smoking and occurrence of lung cancer

**DOI:** 10.1371/journal.pone.0222552

**Published:** 2020-02-25

**Authors:** Juan M. Cubillos-Angulo, Eduardo R. Fukutani, Luís A. B. Cruz, María B. Arriaga, João Victor Lima, Bruno B. Andrade, Artur T. L. Queiroz, Kiyoshi F. Fukutani

**Affiliations:** 1 Instituto Gonçalo Moniz, Fundação Oswaldo Cruz, Salvador, Bahia, Brazil; 2 Faculdade de Medicina, Universidade Federal da Bahia, Salvador, Bahia, Brazil; 3 Multinational Organization Network Sponsoring Translational and Epidemiological Research (MONSTER) Initiative, Salvador, Bahia, Brazil; 4 Curso de Medicina, Faculdade de Tecnologia e Ciências, Salvador, Bahia, Brazil; 5 Universidade Salvador (UNIFACS), Laureate Universities, Salvador, Bahia, Brazil; 6 Escola Bahiana de Medicina e Saúde Pública (EBMSP), Salvador, Bahia, Brazil; King Saud University, SAUDI ARABIA

## Abstract

**Background:**

Cigarette smoking is associated with an increased risk of developing respiratory diseases and various types of cancer. Early identification of such unfavorable outcomes in patients who smoke is critical for optimizing personalized medical care.

**Methods:**

Here, we perform a comprehensive analysis using Systems Biology tools of publicly available data from a total of 6 transcriptomic studies, which examined different specimens of lung tissue and/or cells of smokers and nonsmokers to identify potential markers associated with lung cancer.

**Results:**

Expression level of 22 genes was capable of classifying smokers from non-smokers. A machine learning algorithm revealed that *AKR1B10* was the most informative gene among the 22 differentially expressed genes (DEGs) accounting for the classification of the clinical groups. *AKR1B10* expression was higher in smokers compared to non-smokers in datasets examining small and large airway epithelia, but not in the data from a study of sorted alveolar macrophages. Moreover, *AKR1B10* expression was relatively higher in lung cancer specimens compared to matched healthy tissue obtained from nonsmoking individuals. Although the overall accuracy of *AKR1B10* expression level in distinction between cancer and healthy lung tissue was 76%, with a specificity of 98%, our results indicated that such marker exhibited low sensitivity, hampering its use for cancer screening such specific setting.

**Conclusion:**

The systematic analysis of transcriptomic studies performed here revealed a potential critical link between *AKR1B10* expression, smoking and occurrence of lung cancer.

## Introduction

Worldwide, cigarette smoking is a life-style habit of approximately 1.1 billion individuals and is associated with more than 6 million deaths annually [1]. The immunological responses in persons chronically exposed to smoke from cigarettes are characterized by protracted secretion of inflammatory factors and by accumulation of several leukocytes in lung tissue and production of pro-fibrotic mediators such as transforming growth factor (TGF)-β [2, 3]. These inflammatory perturbations likely result in increased risk development of tobacco associated morbidity including several types of cancer [4], autoimmune disorders [5], chronic obstructive pulmonary diseases [6] and respiratory infections [7].

The role of tobacco smoking in the induction of disturbances in cell/tissue homeostasis and gene mutations, broadly or specifically associated with several types of tumors, have been investigated. Smoking-related malignancies have been reported to be associated with DNA methylation [8] and mutations in several proto-oncogenes, such as *p53*, *KRAS*, *BRCA-1*, *BRCA-2*, *GPX2*, *GABP*, *TCF3*, *CRX*, *CYP2A13*, *CYP2A6*, *CYP2B6*, among others [9–12]. In addition, it has also been reported that components of cigarette smoking modulate immune cell functions, which could lead to loss of T-cell proliferation and antibody responses [13]. Furthermore, chromosomal instability, epigenomic alterations and several mutations have been reportedly associated with lung cancer in particular [14]. Thus, in general, all of these events ultimately culminate with altered gene expression, even though the conversion of carcinogens to DNA adducts is more efficient in some individuals than in others [15]. Therefore, understanding the expression of these genes is important to fully understand the link between smoking exposure and risk of cancer development.

Identification of genetic markers predictive of cancer development is of utmost importance for promoting personalized medicine [16]. Such markers could be implemented as screening strategy for patients who exhibit strong risk factors for cancer, such as cigarette smoking. To identify such potential markers, we performed a systematic analysis of publicly available data from transcriptomic studies performed in lung tissue and/or cells and found that, among most of the studies investigated, increased expression of the gene *AKR1B10* was associated with cigarette smoking as well as lung cancer. Development of a point-of-care assay to assess *AKR1B10* expression in individuals exposed to cigarette smoking may serve as a relevant tool to identify those with high risk of cancer.

## Methods

### Ethics statement

There were no patients directly involved in the research. The present study used publicly available gene expression data from previous studies to perform a meta-transcriptome analysis. All information given to the research team were de-identified.

### Description of discovery datasets

**Fig 1:**
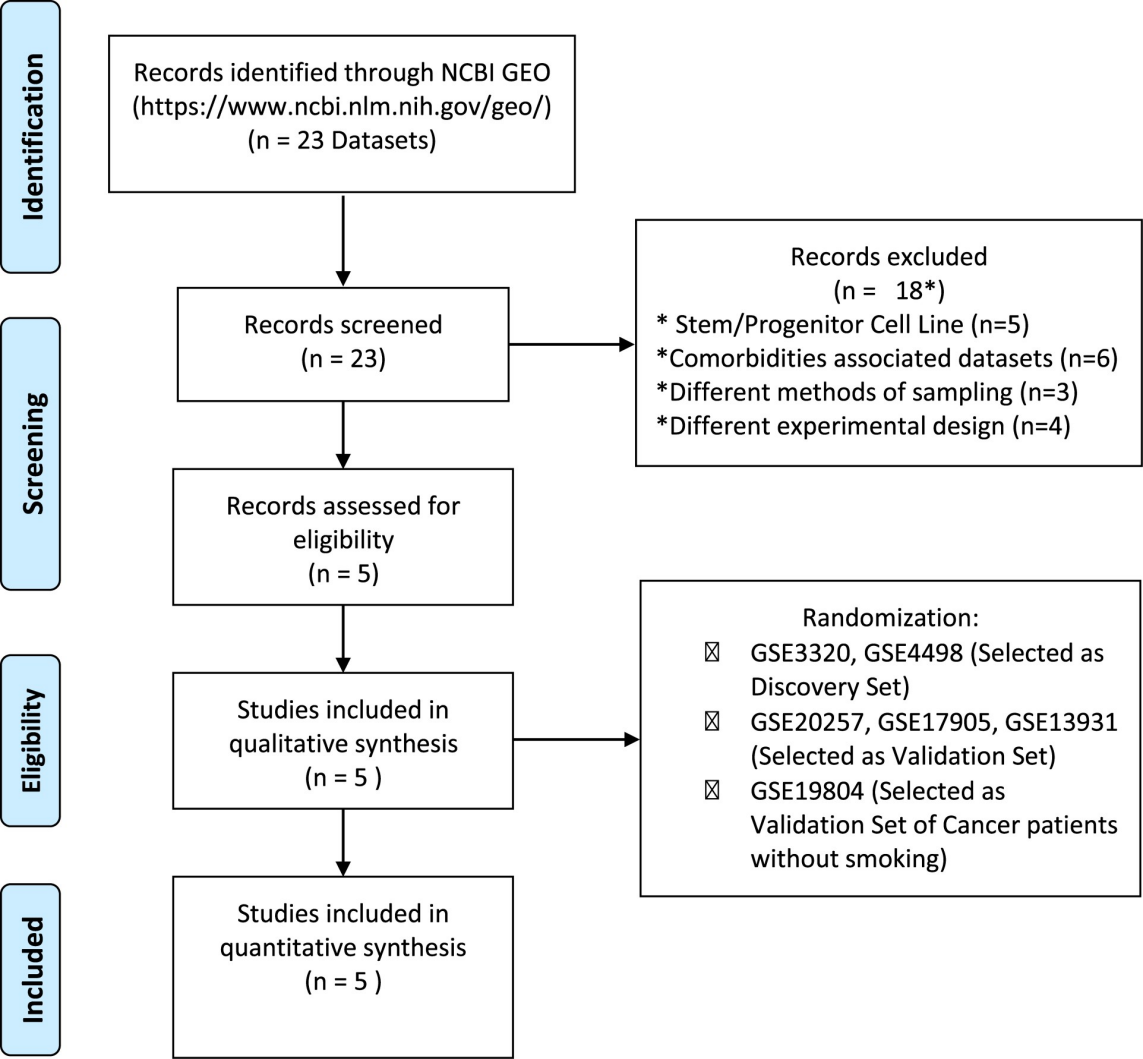
PRISMA flow chart of the microarray meta-analysis. Selection of eligible GEO datasets for systems biology analysis according to PRISMA 2019 flow diagram.

We searched for datasets using the Gene Expression Omnibus (GEO-NCBI -https://www.ncbi.nlm.nih.gov/geo/). The following terms were used: “Smoker”, “Smoking”, “Cigarrete” and “Homo sapiens” and found a total of 23 datasets. We next excluded 18 datasets for a number of reasons listed in Fig 1. Finally, 5 datasets were included. Those datasets were randomized in discovery and validation sets. Similar approach was used to find datasets on lung cancer in nonsmokers (with “nonsmoker”, “nonsmoking”and “cancer” serving as terms used for the GEO search (Fig 1). Thus, using this approach, two previously published microarray datasets were selected to be used as a discovery set (available from the GEO under accession no. GSE4498 [17] and GSE3320 [18]) and 3 have been used as validation set (GSE20257 [19], GSE17905 [20] and GSE13931 [21]). We found other three datasets using gene profiling by array. However, they could not be used for the following reasons: the dataset GSE57048 used mouse cells to measure expression, the GSE124265 used transformed lineage cells and the GSE92662 did not use cigarette-exposed patients. Moreover, there are other datasets by using RNA-seq, however in the present study we have focused on array data only. Due the data distribution differences from each methodology, the direct comparison is difficult. The Dataset GSE4498 [17] was designed with samples of human small airway bronchial epithelium of smokers (n = 10) compared to matched samples from non-smokers (n = 12). The dataset GSE3320 [18] was extracted from samples of human small airway bronchial epithelium to assess gene expression in phenotypically smokers (n = 6) compared to matched non-smokers (n = 5). These included datasets using the same method to collect the samples, by fiberoptic bronchoscopy and brushing. In addition, these studies used a similar transcriptional protocol using the platform Affymetrix Array, making possible to combine both datasets in a discovery set.

### *In silico* validation

We next performed validation of differentially expressed genes detected in the first phase of the investigation using 3 distinct datasets selected by examination of gene expression by smoking status: (i) GSE20257 was published by Shaykhievet al[19]. In this study, they used samples of small airway epithelium collected from individuals who were smokers (n = 51) and also from those who did not smoke (n = 42) and performed an analysis of microarray assays in theses samples. (ii) GSE17905 was published by Wang et al [20]. The authors used large airways samples collected by bronchoscopy of 31 smokers and 21 non-smoker individuals and also performed a microarray analysis. (iii) GSE13931 was published by Carolan et al [21]. The investigators used alveolar macrophages collected by bronchoalveolar lavage of 30 smokers and 19 non-smokers and performed a microarray analysis. (iv) Finally, GSE19804 was available in a publication from Lu et al [22]. This dataset had information of gene expression (assessed by microarray) of 60 pairs of lung cancer tissue and adjacent normal lung tissue from female patients who were not exposed to cigarette smoking.

The datasets were obtained using the *GEOquerry*[23] package and raw expression data of 22 samples present on GSE4498 and 11 samples on GSE3320 were normalized and log2 transformed by *preprocesscoreR* package [24]. Duplicated probes were collapsed by *collapserows* function in *WGCNA* package [25] and all common genes to both datasets were kept and used to merge the datasets. The expression data was submitted to a correction procedure of batch effect using an empirical Bayes framework implemented in the *COMBAT* function available in *SVApackage*[26].

### Statistical analysis

Categorical data were presented as proportions whereas continuous data were plotted as mean and standard deviation (SD). Receiver operator characteristics (ROC) curves were employed to test the accuracy of 22 Genes signature values and AKR1B10 alone to distinguish smokers from those who not a smoker. The differentially expressed genes (DEGs) were identified by applying the absolute ≥1.0log2-fold-change threshold and p-value corrected with FDR adjustment for multiple testing (FDR = 5%), from *limma*package [27]. A volcano plot we used to identify changes in gene expression, the significance versus fold-change on the y and x axes, respectively. We use Venn diagrams to visualize all possible logical relations between all the DEGs between smokers and non-smokers in all datasets evaluated. The modular analysis was performed using the *Cemitool* package [28]. It is based on Weighted correlation network analysis (WGCNA) and default parameters was employed (Beta Parameter = 7). The module annotation was performed with the Kegg database v6.2 [29] and *Gene Set Enrichment Analysis* (*GSEA*) algorithm is available internally in the *Cemitool*package and the Single sample Gene Set Enrichment analysis (ssGSEA) was performed with *GSVA* package [30]. The significant and annotated pathways were clustered using Euclidean distance as dissimilarity measure and average linkage for between-cluster separation (*hclust* function in the stats package in R 3.2.2). All The heatmap was generated in R via the *heatmap*.*2* function from the *gplots* package, using the “scale = “row” switch to Z-score standardize the rows [31]. PCA was performed in order to compare and visualize the expression values of all genes to estimate the variance of the global gene expression with the function *prcomp* a native package in R. The decision trees were employed to validate and identify the minimal gene set that correctly classifies the smokers from nonsmokers from the 22-gene signature [32]. To estimate the decision tree models accuracy, we performed a 10-fold cross validation. The partition procedure was applied to avoid bias in the training/test sets sampling. Thus, the training set was used to tune the parameters, learning and building a model. The validation set was used to test the classifier performance. The sensibility and specificity were measured from the confusion matrix and visualized in the receiver–operating characteristic curve (ROC) [32]. Accuracy was evaluated by area under the curve of ROC plot.

## Results

### Meta-transcriptome signature of smoking

Two expression datasets for smoking were obtained with the accession number of GSE4498 [17] and GSE3320 [18]. Moreover, three datasets have been used as validation set (GSE20257 [19], GSE17905 [20] and GSE13931 [21]. The demographic characteristics of the study participants in each study are described in Table 1.

**Table 1:** Clinical and demographic characteristics of the study participants included in each dataset evaluated.

Characteristics	Smoking datasets					p-value	Cancer dataset
	Discovery datasets		Validation datasets				
	GSE3320	GSE4498	GSE20257	GSE17905	GSE13931		GSE19804
Age, mean (SD)	36.8 (5.6)	43.0 (6.1)	43.6 (9.9)	42.4 (8.6)	42.0 (7.0)	0.1549	61.2 (10.2)
Gender, Male, n (%)	7 (63.6%)	17 (77.3%)	95 (70.3%)	107 (68.2%)	73 (75.3%)	0.6995	0 (0.0%)
Ethnic, n (%)						0.9476	
Black	4 (36.4%)	11 (50.0%)	67 (49.7%)	86 (54.8%)	56 (57.7%)		0 (0.0%)
White	5 (45.5%)	9 (40.9%)	44 (32.6%	46 (29.3%)	32 (33.0%)		0 (0.0%)
Hispanic/Latino	2 (18.2%)	2 (9.1%)	21 (15.5%)	21 (13.4%)	10 (10.3%)		0 (0.0%)
Afro-Hispanic	0 (0.0%)	0 (0.0%)	1 (0.7%)	2 (1.3%)	0 (0.0%)		0 (0.0%)
Asian	0 (0.0%)	0 (0.0%)	2 (1.5%)	2 (1.3%)	0 (0.0%)		60 (100.0%)
Smoke status, n (%)						0.6102	
non-smoker	5 (45.5%)	12 (45.5%)	53 (39.3%)	67 (42.7%)	38 (39.2%)		0 (0.0%)
smoker	6 (54.5%)	10 (54.5%)	59 (43.7%)	90 (57.3%)	60 (61.9%)		0 (0.0%)
COPD, n (%)	0 (0.0%)	0 (0.0%)	23 (17.8%)	0 (0.0%)	0 (0.0%)		0 (0.0%)
Lung Cancer, n (%)	0 (0.0%)	0 (0.0%)	0 (0.0%)	0 (0.0%)	0 (0.0%)		60 (100.0%)

COPD: Chronic obstructive pulmonary disease.

**Fig 2:**
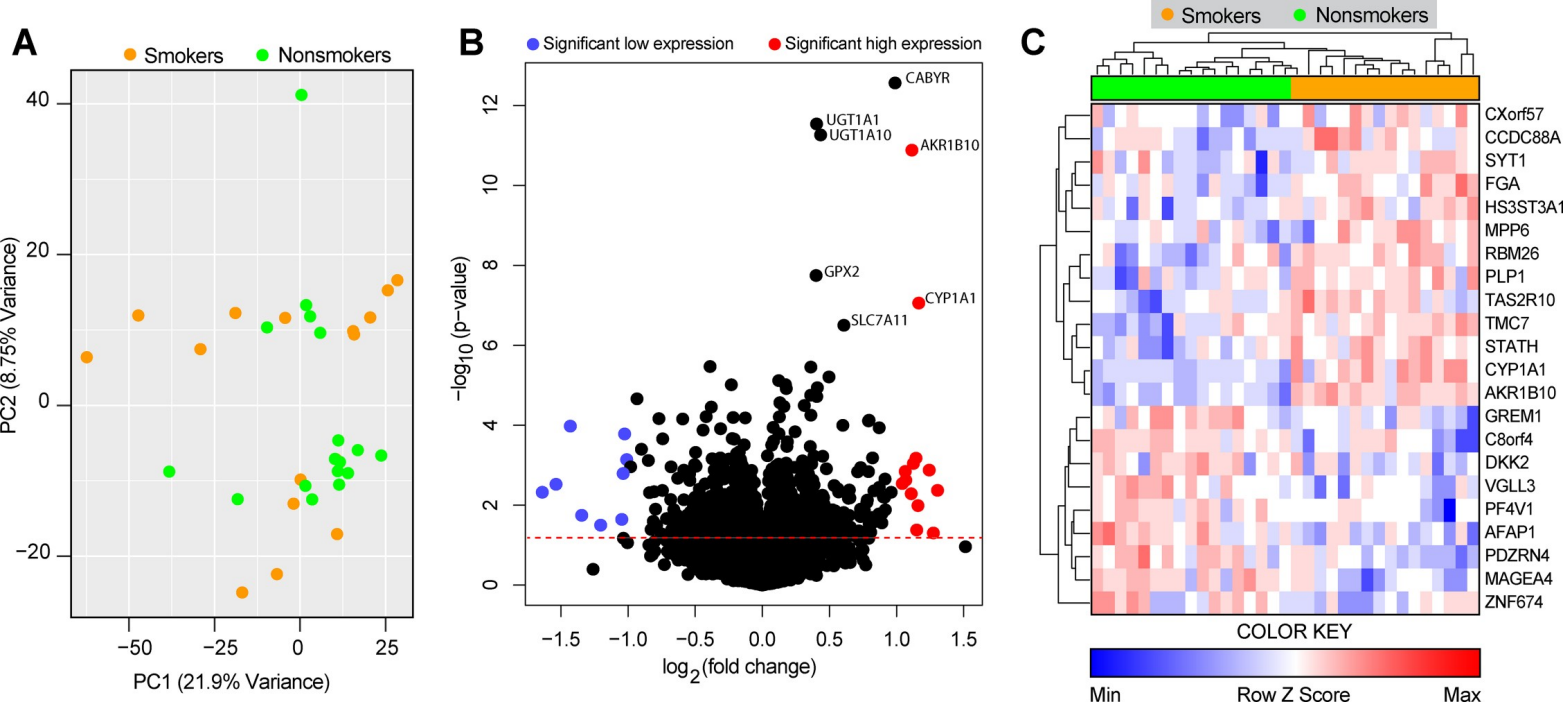
Differentially expressed genes associated with cigarette smoking. We analyzed publicly available data of 2 datasets of small airways transcriptome (RNAseq). (A) A principal component analysis (PCA) model of 13,516 genes was used to distinguish smokers from nonsmokers. (B) Volcano plot of all genes (smoker vs. nonsmokers). (C) 22 differentially expressed genes (DEGs), defined as p<0.05 after 1%FDR and 1.0-fold change expression, were found and together were able to discriminate the clinical conditions.

After preprocessing and merging the datasets, we applied a Principal Component Analysis (PCA) algorithm using the expression values of all genes to estimate the variance of the global gene expression. This analysis revealed that the subgroups of smokers and non-smokers could not be separated, and 2 main groups containing both smoker and non-smoker individuals were observed (Fig 2A). To visualize the overall profile of individual gene expression, we used a volcano plot (Fig 2B). This approach indicated presence of a total of 800 statistically significant genes (p<0.05, corrected by Benjamini–Hochberg false discovery rate [FDR]), of which 375 genes were upregulated and 425 genes were downregulated (Fig 2B). Additional analyses identified 22 the differentially expressed genes (DEGs), defined here and genes which exhibited more than ± 1-fold-difference variation (smokers vs. non-smokers) and a significant p-value after FDR adjustment (p<0.05). Such DEGs were inputted in an unsupervised two-way hierarchical clustering analysis. The results demonstrated that when considered together, the 22-gene signature was capable of classifying smokers from non-smokers into completely separate clusters (Fig 2C).Moreover, using canonical discriminant models to further characterize the association of all 22 genes signatures used the validation set GSE20257 [19], GSE17905 [20] and GSE13931 [21]. The area under the ROC curve (AUC) for GSE17905 [20] was 0.86 (P<0.0001), for GSE20257 [19] AUC was 0.86 (P<0.0001) and GSE13931 [21] was 0.60 (P = 0.4236). The ROC curve analyses are summarized in Table 2. This table presents the overall accuracy, sensitivity and specificity of the DEGs identified in human small airway bronchial epithelium (GSE20257 and GSE17905) and in alveolar macrophages (GSE13931).To answer whether sex, age and ethnicity had any influence in the overall gene expression profiles, we performed a Principal Component Analysis (S1 Fig) using both the discovery datasets and the three independent validation sets. Using this approach, we found that such demographic characteristics were not associated with unique expression profiles.

**Table 2:** Detailed information obtained from the ROC curve analysis used in the study.

Dataset	Tissue	Genes/signature	AUC	95% CI	p-value	Sensibility	95% CI	Specificity	95% CI
GSE17905*	Small and large airway bronchial epithelium	22-gene	0.864	0.808–0.989	<0.0001	83.87	66.2%-94.5%	95.24	76.1%-99.8%
GSE20257*	Small airway bronchial epithelium	22-gene	0.862	0.845–0.973	<0.0001	69.05	52.9%-82.3%	98.04	89.5%-99.9%
GSE13931*	Alveolar Macrophages	22-gene	0.607	0.396–0.740	0.4236	80.00	61.4%-92.2%	42.11	20.2%-66.5%
GSE19804**	Lung tissue	AKR1B10	0.760	0.720–0.880	<0.0001	35.00	23.1%-48.4%	98.31	90.9%-99.9%

*Smokers versus nonsmokers comparison

**Cancer versus non cancer comparison

**Fig 3:**
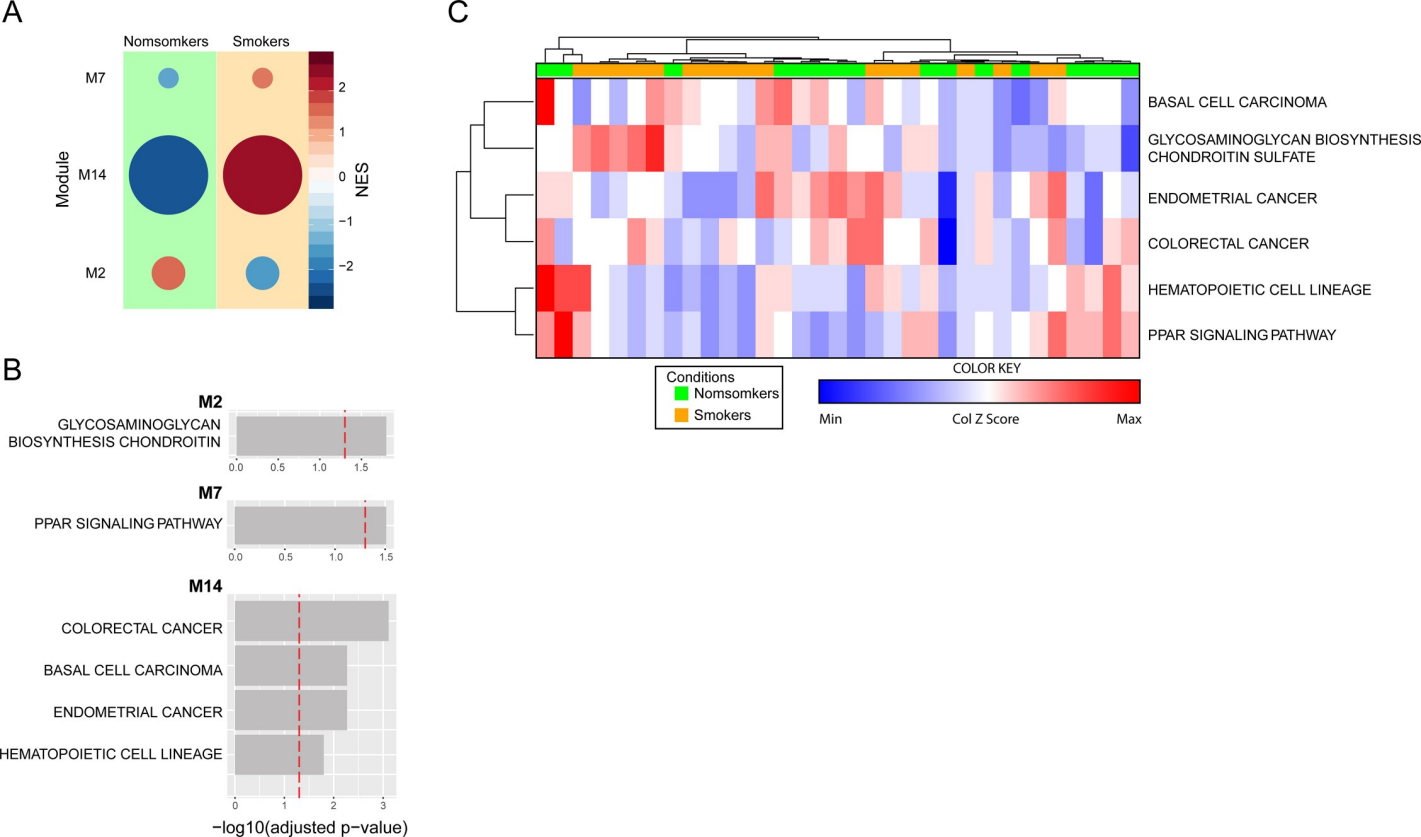
Gene pathway analysis in smokers and nonsmokers. (A) Co-expressed modules of all genes. Circle sizes are proportional to the normalized enrichment scores (NES). (B) The modules were annotated using Keg package for R. Dashed lines represent significance threshold. (C) Hierarchical cluster analysis (Ward’s method) using the NES scores for each annotated module and calculated for each person was employed test discrimination between smokers and nonsmokers.

To delineate the gene pathways from which the overall transcription profile in smokers vs. non-smokers were involved, we used the *CemiTool* package [28]. We detected 3 distinct co-expressed gene modules, annotated in Kyoto Encyclopedia of Genes and Genomes (*Kegg)* database (Module [M] 2, M7, M14) (Fig 3A). Two modules were enriched in the non-smoking samples compared to smokers, based on the normalized enrichment scores (NES). The first module (M2), found to be enriched in non-smokers was Glycosaminoglycan biosynthesis chondroitin (log10 p = 1.57). A second module (M7) was overrepresented in smokers compared to non-smokers and showed to be enriched in the Peroxisome proliferator-activated receptor (PPAR) signaling pathway (log10 p-value = 1.5). A third module, also more representative in smokers encompassed colorectal cancer (log10 p-value = 3.2) and basal cell carcinoma (log10 p-value = 2.4) (Fig 3B). We next calculated the NES for each top ranked pathway identified per individual study subject and found that, when considered together, such pathways were not able to cluster smokers and non-smokers separately (Fig 3C).

### A 22-gene signature in lung tissue, but not in alveolar macrophages, including *AKR1B10* as the most informative marker, discriminates smoking from nonsmoking individuals

**Fig 4:**
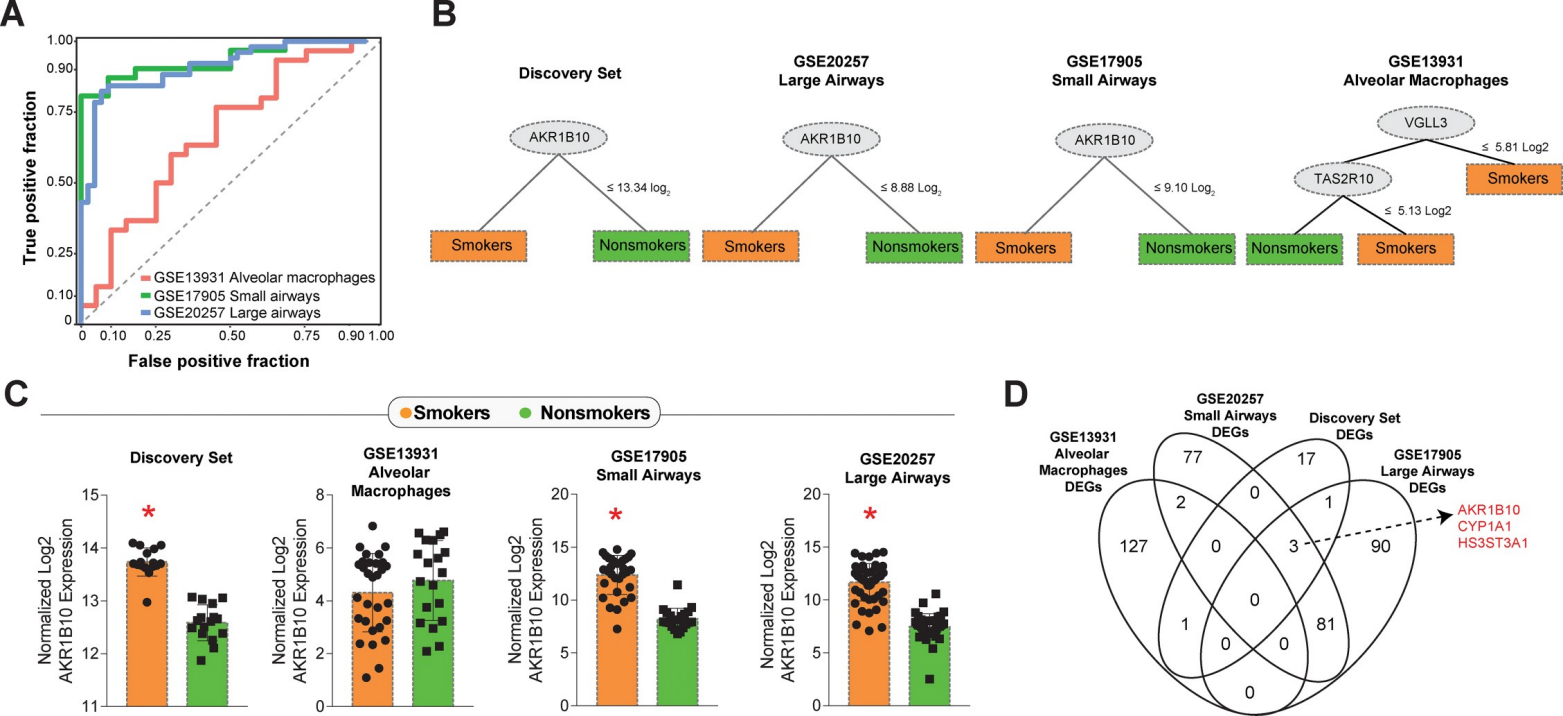
Defining the molecular signatures of smoking. (A) Data on the 22 DEGs found in our discovery analyses were used to validate discrimination between smokers and nonsmokers in 3 different previously published datasets. (B) Machine-learning decision trees were built for each dataset to describe the most relevant genes driving discrimination. Of note, the gene *AKR1B10* was found to be the main discriminator in 3 out of the 4 datasets examined. (C) Scatter plots of the *AKR1B10* gene expression in the 4 datasets. (D) Venn diagram of the DEGs in each dataset shows *AKR1B10* in the intersection of 3 datasets extracted from lung tissue specimens but not included among DEGs from alveolar macrophages. *p<0.05 (Student’s t-test).

To validate our discoveries, we tested the 22 DEGs identified in our analyses in 3 distinct datasets that compared smokers and non-smokers: (i) GSE20257, that was composed by data from small airways samples, (ii) GSE17905, which compared gene expression from large airways samples and (iii) GSE13931, which used data from alveolar macrophages. Discriminant analyses using Receiver Operating Characteristic (ROC) curves were able to reveal high accuracy of such gene signature to distinguish smokers from nonsmokers in the 2 datasets that large and small airway samples (GSE20257 Area under the curve [AUC]: 0.862, p<0.0001; GSE17905 AUC: 0.864, p<0.0001). The same approach indicated that when a dataset from alveolar macrophages was considered, the 22-gene signature was not able to distinguish the study groups (GSE13931AUC: 0.607, p = 0.423) (Fig 4A). We next employed a machine-learning approach using decision trees to identify which markers from the 22-gene signature would exhibit more robust discrimination power in each dataset evaluated. Of note, the gene *AKR1B10* was the most informative gene in the discovery set and also in the 2 distinct datasets that used large or small airway tissue (Fig 3B). In the dataset that used gene expression values form alveolar macrophages, *AKR1B10* was not shown to be relevant in discrimination, and a combination of 2 other genes (*VGLL3* and *TAS2R10*) accounted for the differences between smokers and non-smokers (Fig 4B). *AKR1B10* expression was higher in smokers compared to non-smokers in all datasets evaluated, except again in the GSE13931, which used data on alveolar macrophages (Fig 4C). Furthermore, we plotted Venn diagrams of all the DEGs between smokers and non-smokers in each dataset to verify overlaps. We confirmed that *AKR1B10* was a DEG commonly shown in the discovery set as well as in the databanks which used airway tissue samples, but not in the alveolar macrophage dataset (Fig 4D). The 2 other DEGs found in smokers were *CYP1A1* and *HS3ST3A1* (Fig 4D). *CYP1A1* encodes a protein that localizes at the endoplasmic reticulum and its expression is induced by polycyclic aromatic hydrocarbons, some of which are found in cigarette smoke [33]. *HS3ST3A1*is a member of the heparan sulfate biosynthetic enzyme family [34].

### Testing *AKR1B10* as a potential biomarker of lung cancer in patients who do not smoke

**Fig 5:**
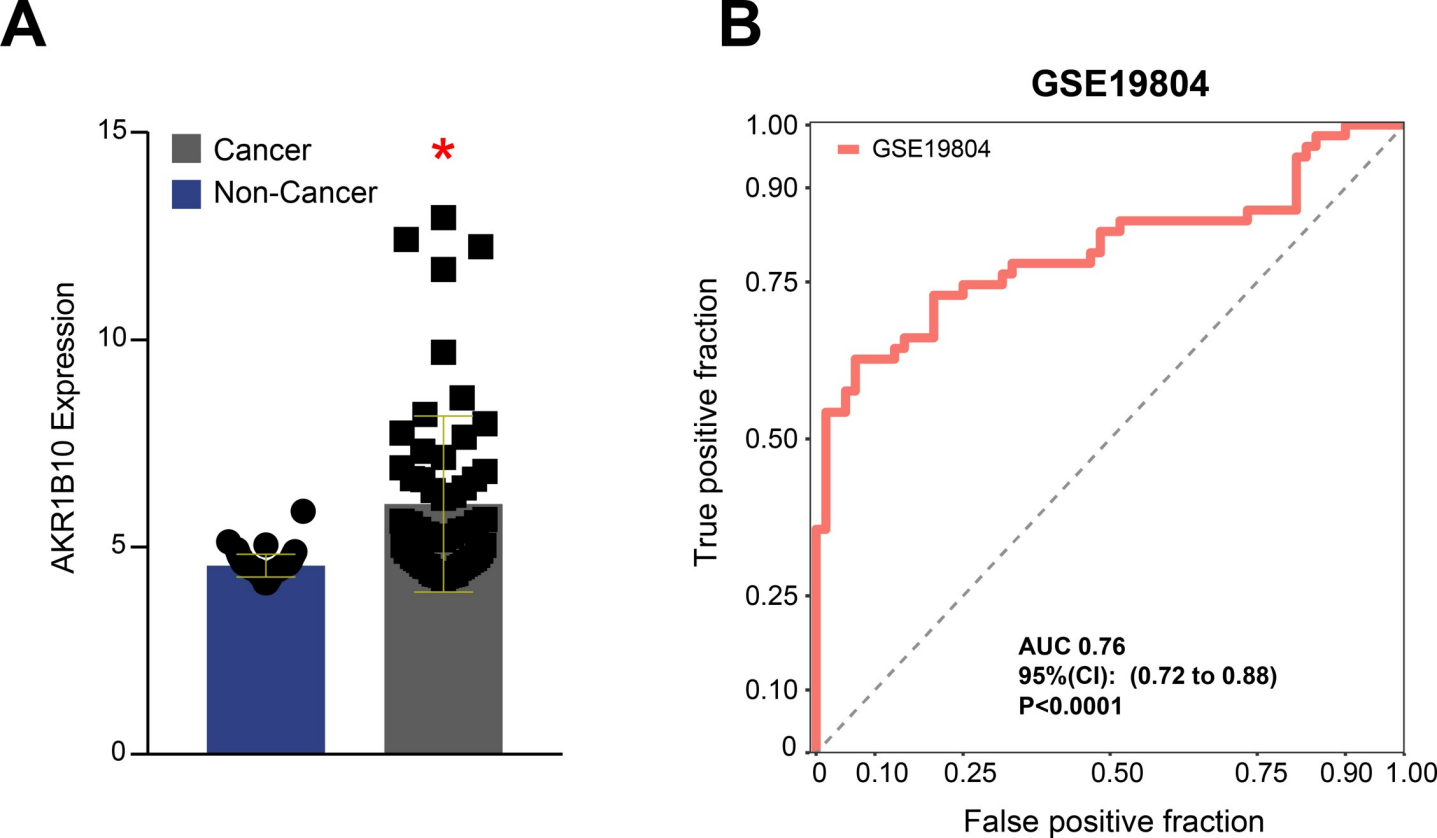
In nonsmokers, higher *AKR1B10* expression is detected in lung cancer. (A) We analyzed *AKR1B10* gene expression values in a published dataset of neoplastic lung tissue microarray in nonsmoking individuals who were diagnosed with lung cancer and compared to ipsilateral healthy lung tissue specimens (controls.) Scatter plots of *AKR1B10* gene expression in the groups. *p<0.05 (Student’s t-test). (B) Receiver Operator Characteristics (ROC) indicated a high accuracy to discriminate cancer tissue from controls.

The results described above demonstrate that higher *AKR1B10* expression hallmarks tissue airways from smokers. Smoking is a well-established risk factor for lung cancer [9]. We next tested whether AKR1B10 gene expression could also be useful to inform presence of cancer in the absence of exposure to smoking. We downloaded the dataset GSE19804, which included tissue samples from non-small cell lung cancer as well as ipsilateral healthy lung tissue obtained from patients who did not present history of cigarette smoking. The *AKR1B10* gene expression was substantially higher in the specimen collected from the tumor compared to the healthy lung tissue in the same patients (Fig 5A). ROC curve analysis indicated that *AKR1B10* gene expression value was able to correctly identify non-cancer tissue (AUC 0.76, P<0.0001), with relatively high specificity (98.31%). Nevertheless, the results demonstrated low sensitivity (35%), which limits the use of such biomarker for screening in the clinical setting (Fig 5B and Table 2).

## Discussion

In the present study, we examined a number of publicly available transcriptome data to identify a 22-gene signature that could distinguish lung tissue specimens from smokers vs. non-smoking individuals. The most relevant finding of the initial part of analyses using the 22-gene signature was the *AKR1B10* expression level was the most informative in such discrimination among 3 different datasets obtained from lung tissue, but not in the transcriptome data originated from alveolar macrophages. Moreover, ROC curve analysis indicated that *AKR1B10* gene expression level exhibited high specificity to but low sensitivity to identify neoplastic from healthy lung tissue in persons not exposed to cigarette smoking. Such analysis however revealed that the overall accuracy is below 80%, and thus not an ideal biomarker for diagnostic purposes. Nevertheless, these findings are important because they have identified *AKR1B10* as a biomarker which expression is triggered by cigarette smoking and can be simultaneously observed in lung cancer specimens. It is possible that such gene may be involved in carcinogenesis associated with cigarette smoking. In fact, among the multiple carcinogens from cigarette smoke, the nitrosamine 4-(methylnitrosamino)-1-(3-pyridyl)-1-butanone (NNK) is described to play a critical role in lung carcinogenesis [35]. Carbonyl reduction takes place in both microsomal and cytosolic fractions from different human tissues such as lung and liver [36]. Within these subcellular fractions, several enzymes have been described to mediate NNK reduction, including the protein encoded by *AKR1B10*, which is from the aldo-keto reductase superfamily (AKR) [37]. Our findings suggest an association between *AKR1B10* and smoking, however, the direct relationship with occurrence of lung cancer was not completely validated here. Moreover, if validated in other settings, this gene could be suitable to be used as rule-out test in which non-smoking individuals presenting low *AKR1B10* expression would have low risk of having lung cancer. Additional studies are warranted to directly test this hypothesis.

The gene *AKR1B10* found differentially expressed in lung tissue from smokers vs. non-smokers has been previously described in experimental studies to play an important role in the pathophysiology of lung cancer [38]. *AKR1B10* is a regulator of the synthesis of fatty acid and participates in the metabolic pathway of lipids and isoprenoids [39]. In addition, the protein encoded by *AKR1B10* exhibits a high retinaldehyde reductase activity [40]. Importantly, *AKR1B10* can metabolize specific substrates, such as aldo-ketoreductases; farnesal, geranylgeranil, retinal and carbonyls [41]. Such activity is associated with promotion of carcinogenesis [42]. *AKR1B10* has also been shown to promote cancer cell survival by 2 distinct studies [43, 44]. These previous investigations revealed that knocking down *AKR1B10* expression induces cancer cell apoptosis and inhibited cancer cell proliferation, suggesting *AKR1B10* could serve as a potential therapeutic target.

Aside from being associated with lung carcinogenesis, *AKR1B10* expression has also been linked to the development of several additional types of cancers. In hepatocellular carcinoma (HCC), *AKR1B10* expression is found upregulated, and experimental deletion of such gene inhibited the proliferation of HCC cells tumor growth in a xenograft mice model [45]. In HCT-8, a human colon adenocarcinoma cell line, and NCI-H460, a human lung carcinoma cell line, *AKR1B10* gene deletion has been shown to induce cell apoptosis and mitochondrial degeneration, leading to oxidative stress [43]. Furthermore, higher *AKR1B10* expression has been observed in squamous cell lung carcinoma (SCC) associated with smoking [46]. Finally, our findings indicate that *AKR1B10* is overexpressed in lungs of healthy people who smoke but had no cancer as well as in lung carcinoma from non-smokers. These observations argue that cigarette smoking already modifies the microenvironment of the lung epithelium probably creating a favorable scenario for carcinogenesis. This idea corroborates with previously published studies which demonstrated that smoking per se mediates upregulation of *AKR1B10* expression in the airway epithelia of healthy smokers with no evidence of lung cancer [47]. Thus, there is strong evidence to suggest that cigarette smoking-induced upregulation of *AKR1B10* may represent an initial critical step in the cascade of events leading to lung cancer.

In addition to *AKR1B10*, our analysis revealed that 2 additional genes, *CYP1A1* and *HS3ST3A1*, overlapped in the datasets as DEGs capable of discriminating smokers from nonsmokers. Of note, *CYP1A1* has also been described to induce carcinogenesis, by promoting CYP‐catalyzed epoxidation reactions, resulting in the formation of reactive metabolites that can cause DNA [48, 49]. Moreover, *CYP1A1* polymorphisms in smokers increase susceptibility to stomach cancer [50]. Furthermore, *HS3ST3A1*gene encodes the enzyme 3-O-sulfotransferase, which catalyzes the biosynthesis of a specific subtype of heparan sulfate (HS), 3-O-sulfated heparan sulfate, which is found to be upregulated in human lung cancer specimens and to contribute to its elevated metastatic potential [34]. Thus, the 3 genes found commonly differentiate regulated in individuals exposed to cigarette smoking are all known to favor development of cancer and could be used as an early biomarker of disease progression in high risk populations, but future studies specifically designed to test this hypothesis are necessary.

Our study has several strengths such as the large number of samples evaluated, the use of discovery and validation datasets using different lung tissue/cellular types and different clinical conditions. An important limitation was the low number of studies included, which was dependent on publicly available datasets. In addition, we have not performed validation in experimental systems. Regardless, by performing a systematic analysis of publicly available data from transcriptomic studies of lung tissue and cells, our study provides strong evidence to support a potential role of *AKR1B10* in smoking-associated lung cancer.

## Supporting information

S1 Fig Principal component analysis testing influence of demographic characteristics in the overall expression profiles. A principal component analysis (PCA) was employed to test whether the sex, ethnicity and age could cluster patients in the two discovery datasets (GSE4498 [17] and GSE3320 [18]) and in the three validation sets separately (GSE20257 [19], GSE17905 [20] and GSE13931 [21]).
(TIF)
