# Supplementary figures and images for: Systems biology analysis of publicly available transcriptomic data reveals a critical link between AKR1B10 gene expression, smoking and occurrence of lung cancer
Source: PLoS One. 2020 Feb 25;15(2):e0222552. doi: 10.1371/journal.pone.0222552 (PMC7041805; doi:10.1371/journal.pone.0222552)

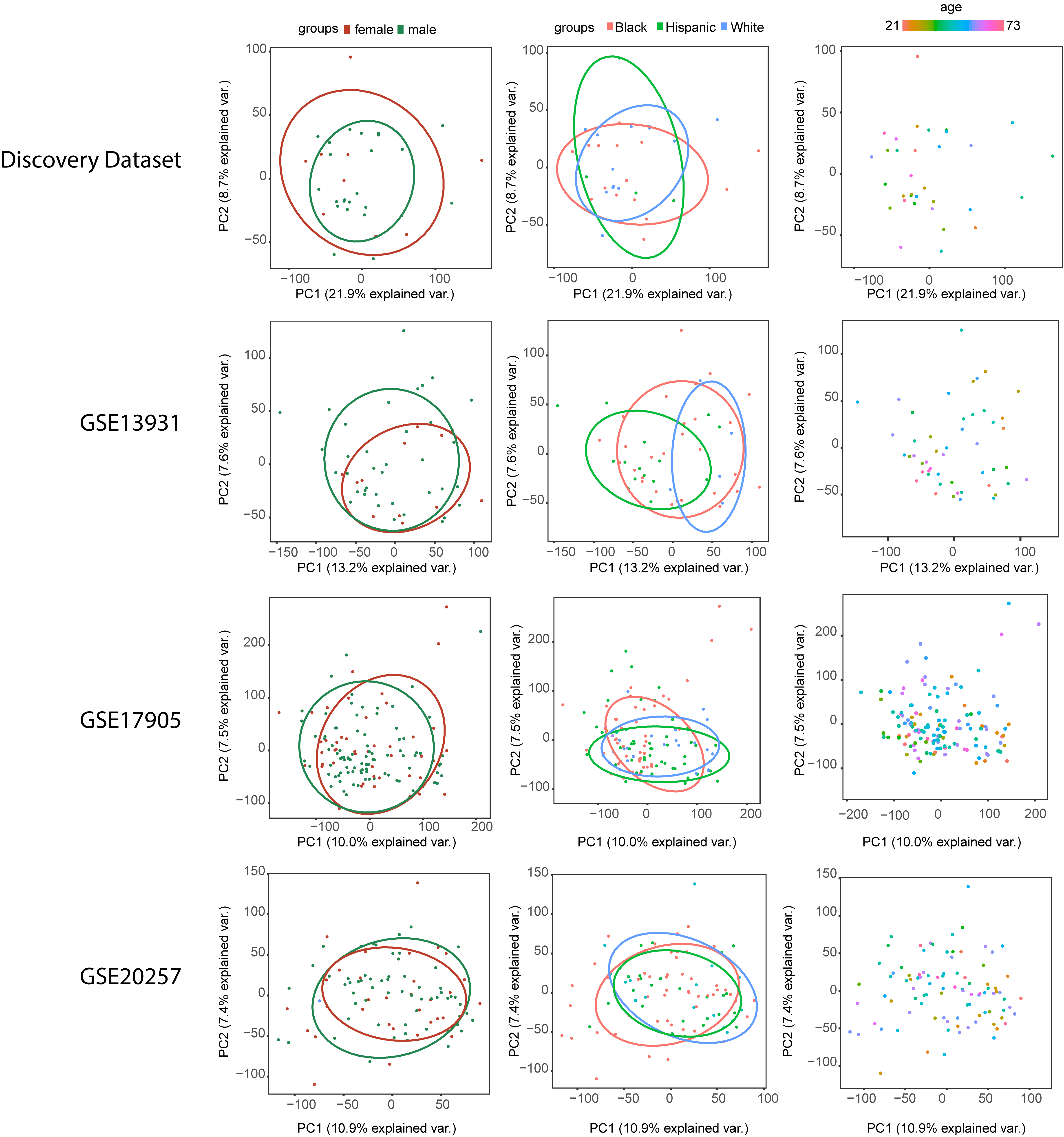

Supplement: S1 Fig — A principal component analysis (PCA) was employed to test whether the sex, ethnicity and age could cluster patients in the two discovery datasets (GSE4498 [17] and GSE3320 [18]) and in the three validation sets separately (GSE20257 [19], GSE17905 [20] and GSE13931 [21]). (TIF) [file pone.0222552.s001.tif]
